# The interaction between drought stress and nodule formation under multiple environments in chickpea

**DOI:** 10.1371/journal.pone.0276732

**Published:** 2022-10-27

**Authors:** Tawffiq Istanbuli, Ahmed Abu Assar, Sawsan Tawkaz, Tapan Kumar, Alsamman M. Alsamman, Aladdin Hamwieh

**Affiliations:** 1 International Center for Agricultural Research in the Dry Areas (ICARDA), Beirut, Lebanon; 2 Department of Oil Crops, Agriculture Research Center (ARC), Wad Madani, Sudan; 3 International Center for Agricultural Research in the Dry Areas (ICARDA), Amlaha, India; 4 Agricultural Genetic Engineering Research Institute, Agricultural Research Center, Giza, Egypt; CSIR - Institute of Himalayan Bioresource Technology, India, INDIA

## Abstract

Environmental stresses, particularly drought, limit symbiotic nitrogen fixation in legumes, resulting in decreased yielding capacity. Drought is one of the most important constraints limiting yield potential in crops and it is the major abiotic stress that can cause more than 70% yield loss in chickpea. In this study, a total of two hundred four chickpea (*Cicer arietinum* L.) genotypes were selected to study the interaction between drought stress and nodule formation. This interaction was assessed by using morphological, yield and yield components. The field experiments were laid out in two locations (Terbol and Kfardan stations, Bekaa valley, Lebanon) using Alpha lattice design with two replications and two watering treatments (irrigation and rainfed) during 2016 and 2017 seasons. Parameters that were measured include days to 50% flowering (DFL), day to maturity (DM), plant height (PLH), nodule biomass (NB), nodule fresh weight (NFW), nodule dry weight (NDW), grain yield (GY), Biological yield (BY), 100 seed weight (100SW) and drought tolerance stress (DTS). The results indicated a significant variation between genotypes, environments and other morphological, yield and yield components traits. Drought stress reduced significantly the yield and the nodule’s characteristics, biological and grain yield. The genotypes with the highest levels of drought tolerance, such as IG70399, IG8256, IG71832, IG70270, and IG70272, showed a minimal decrease in yield and nodule biomass. Nodule observations significantly and positively correlated with GY (0.36-0.38) under drought stress treatment. The correlation values for nodule characteristics with DFL and DM were higher under drought stress compared to irrigated conditions. This is a comparative study between drought stress and nodule formation traits associated with morphological, yield and yield components traits.

## Introduction

Chickpea (*Cicer arietinum L.*) is the most economically important food legume crops It is among the world’s three most important pulses with over 17.22 million tons of chickpea was produced on 17.85 million hectares with 964.6 kg/ha productivity per unit area in 2018 [1]. It is a self-pollinated diploid (2n = 16)with a genome size of 738 Mbps [2]. Chickpea is cultivated in more than 50 countries, especially in South Asia and sub-Saharan Africa [3], which have considerable importance as food, feed and fodder [4]. About 90% of world’s chickpea is grown under rain-fed conditions and experiences terminal drought stress during their productive phase resulting in heavy yield losses accounting for 3.4 million hectares [5].

Chickpea is a rich source of protein and starch in the growing countries [3], the seeds contain 20–30% protein and approximately 40%carbohydrates. Moreover, being a grain legume, it plays an integral part in diversifying the cereal-based cropping system because of its ability to add 60–103 kg/ha of nitrogen to the soil through symbiotic nitrogen fixation [6]. Declining soil fertility, loss of organic matter, inappropriate use of water resources, excessive use of fertilizers, increasing soil acidity and salinity of dry lands, all pose real threats to both economic and biological sustainability. Increasing and extending the role of biofertilizers such as Rhizobium can reduce the need for chemical fertilizers and decrease adverse environmental effects.

Chickpea exhibits an important characteristic of fixing atmospheric N_2_ through symbiotic association with compatible Mesorhizobium soil bacteria, the common chickpea-specific rhizobia species. Symbiotic N_2_ fixation is the major route for providing a large nitrogen proportion for human consumption and animal feed and contributes to agriculture sustainability [7]. Thus, chickpea can obtain 60–103 kg/ha of its nitrogen requirement through symbiotic nitrogen fixation (SNF) by fixing 140 kg atmospheric N_2_ ha^-1^ [6]. Symbiotic nitrogen fixation in legumes is limited by environmental stresses, mainly salinity and drought, resulting in decreased yielding capacity [8].

Drought is one of the most important constraints limiting yield potential in cereal and legume crops. It is the major abiotic stress that can cause more than 70% yield loss in chickpea [9, 10]. Chickpea crop responds variably to drought stress depending upon the variety, growth stage, and stress duration [11, 12]. Drought stress affects the chickpea crop at all growth stages. The effects can be estimated from quantitative and qualitative parameters including physiological parameters, biochemical parameters, osmotic regulation, molecular and gene expression regulation, nutrient uptake, nodule formation, yield and yield components [11]. To resist the drought, the plant has morphological, physiological, and biochemical recourse to changes [13]. Therefore, the recognition of drought tolerance mechanisms of legumes is important in order to improve their agronomic performance. Thus, an understanding of SNF responses to drought stress and the identification of factors that affect the rate of SNF in chickpea nodules are crucial for enhancing the productivity of this crop by genetic engineering [7].

## Materials and methods

### Plant material and growth conditions

**Fig 1:**
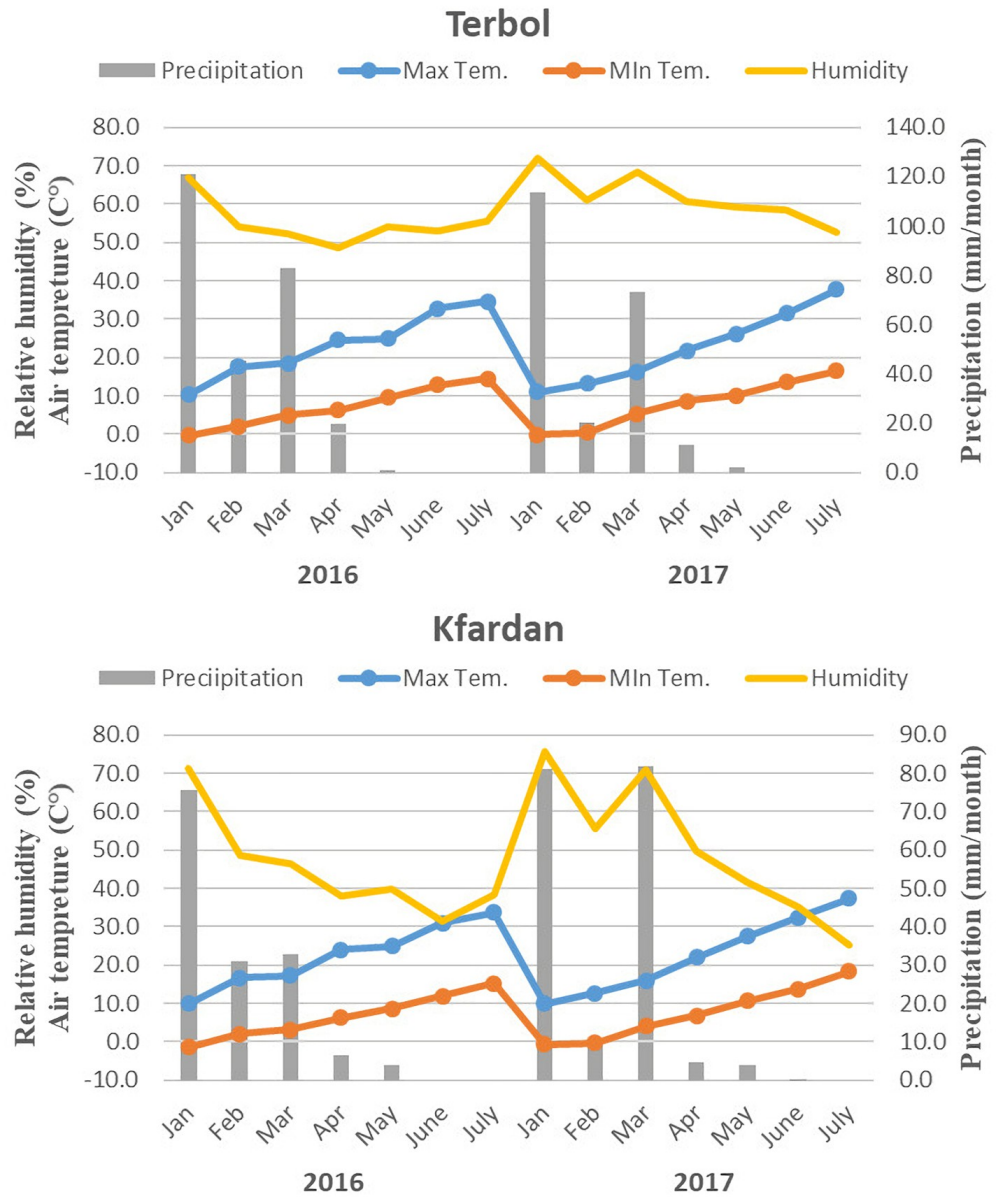
Monthly total precipitation. Maximum and minimum mean air temperature and relative humidity for Terbol and Kfardan stations in 2016, and 2017 seasons.

A total of two hundred four chickpea genotypes, including 199 subset accessions and 5 breeding lines were selected from the genetic resource section (GRS), of ICARDA based on the passport data using focused identification of germplasm strategy (FIGS) for BNF screening in chickpea (S1 Table). The experiments were carried out in two locations in Lebanon (Terbol and Kfardan). Terbol is located at latitude 33° 49 N and longitude 35° 59 E at an altitude of 890 m above the mean sea level. Chemical analysis for Terbol soil by the Lebanese Agriculture Research Institute (LARI), Beqaa, Lebanon showed that the soil is poor in nutrients (N 0.05%, P 7.6 ppm, Fe 4.3 ppm, organic matter 0.9%) and rich in K 380 ppm, Mg 297 ppm with pH 7.8 and the soil texture is clay. While Kfardan soil analysis was poor in nutrients (N 0.05%, P 7.2 ppm, Fe 1.7 ppm, organic matter 0.8%) and rich in K 410 ppm, Mg 725 ppm with pH 6.5 and the soil texture is clay loam. At both locations the soil treated with fertilizer (NPK 15x15x15) to compensate for the lack of elements in the soil. Kfardan is located at latitude 30° 01 N and longitude 36° 03 E at an altitude of 1080 m above the mean sea level. Climatically, the area is placed in the semi-arid temperate zone with cold winter and moderate summer. The total values of evapotranspiration in Terbol 219 mm, 192 mm and Kfardan 121mm, 159 mm during the season 2016 and 2017, respectively. Average rainfall was about 537 mm, 436 mm respectively and most of the rainfall is concentrated between winter and spring (Fig 1). The experiments were planted in two replications with two water treatments (irrigation and rainfed) for two seasons 2016 and 2017 with an Alpha Lattice design, 35cm between rows and 2.5 m row length (25 Plant/row). During the plant season the plants treated with insecticide (Chlorpyrifos 48% EC) and Fungicide (chlorothalonil 37.5%). The field experiments have been numbered to eight environments: (1) Terbol Rainfed-2016, (2) Kfardan Rainfed-2016, (3) Terbol Rainfed-2017, (4) Kfardan Rainfed-2017, (5) Terbol Irrigated-2016, (6) Kfardan Irrigated-2016, (7) Terbol Irrigated-2017, (8) Kfardan Irrigated-2017.

### Morphological traits for chickpea subset

The morphological traits were recorded for days to 50% flowering (DFL), day to maturity (DM), plant height (PLH), nodule biomass (NB), nodule fresh weight (NFW) and nodule dry weight (NDW). DFL and DM were counted from the first day of soil wetting sufficient for germination from each plot. Data for NB, NFW and NDW were taken randomly from three plants from each water treatments, replications and locations at flowering stage. Average NB from three plants were taken as a volume in m^3^. NFW was recorded by removed the nodules immediately from the root and weights (g). The NDW were recorded after oven drying for 2 days (to constant weight) at 48°C.

### Yield and yield components for chickpea subset

At maturity stage, three plants were harvested from the middle of each plot for water treatments, replications, locations in season 2016 and 2017 to determined yield components. The seeds were cleaned and weighed to determine grain yield (GY) as an average of the total seed dry weight (g) for 3 plants/plot. Biological yield (BY) was calculated as an average of the total shoot dry weight (g) for 3 plants/plot. Sub-samples of the seeds were used to determine 100SW. The harvest index was determined as the ratio of grain yield to biomass yield.

### Drought tolerance score (DTS)

The drought tolerance scores (DTS) were designed by ICARDA [14] for the assessment of drought tolerance in chickpea as a score (1–9) at the maturity stage. 1 = free, 2 = highly tolerant, 3 = tolerant, 4 = moderately tolerant, 5 = Intermediate tolerant, 6 = moderately susceptible, 7 = susceptible, 8 = highly susceptible, 9 = 100% death. Details not required.

### Statistical analysis

Data were analyzed with the GenStat program version 19. The field experiments were analyzed for each environment using REML meta-analysis for multi-environments considering genotype as random. Variance components due to genotypes (*σ*^2^_g_) and error (*σ*^2^_e_) and their standard errors were determined. Heritability was estimated as *h*^2^ = *σ*^2^_g_ / (*σ*^2^_g_ + *σ*^2^_e_). Here, the year was treated as a fixed effect and the genotype (G) × environment (E) interaction as random. The significance of the fixed effect of the year was evaluated by the Wald statistic that asymptotically follows a *χ*^2^ distribution. Data were represented by predicted means of 2 replicates ± SEM. A correlation matrix among the DTS, morphological traits and yield components were calculated using the means of each trait across all environments including drought stress and non-drought stress.

## Results

### Weather during crop growth seasons

The weather during the crop growing seasons varied largely in the time of rains and explained the differences in the sowing time. There were overcasts and more rains received in Terbol location during 2016 at the time of growing seasons (second half of March, April and May). About 106.6 mm rain received during 2016 as compared to 61.8 mm during 2017. While the total rains in Kfardan location were 43.2 and 59.6 mm during 2016 and 2017 seasons, respectively (Fig 1). The preceding rainy season rains before sowing (January, February and the first two weeks from March) were 170.6, 158 mm at Terbol location and 106.4, 121.8 at Kfardan location during 2016 and 2017 seasons, respectively, resulting in a fully saturated soil profile at sowing. The air temperatures during growing seasons were not different in the two seasons and locations, while the relative humidity was higher in Terbol than Kfardan locations during growing seasons.

### Variation in morphological traits

The genetic variation for morphological and phenological traits were significant (P < 0.001) in genotypes (G), environments (E) and the interaction (G x E) (Table 1). The overall means for each irrigation treatment across years and locations had shown that irrigation delayed the DFL and the DM (Table 2). Under drought stress condition DFL and DM reduced by 2.3% and 2.2%, respectively. The heritability of 50% flowering was relatively higher in rainfed 0.45 than in irrigated 0.40 treatments. PLH decreased by 22% under drought stress condition, with no differences in heritability under both water treatments. Nodule characteristics decreased with increasing levels of drought stress especially for genotypes IG115380 and IG70309. The reduction of NB, NDW and NFWrelative to irrigated treatment were 17%, 22.3% and 14.7% respectively (Table 1). The heritability for nodule observations were higher in rainfed conditions than in irrigated conditions.

**Table 1:** Components of variance of morphological traits of 204 genotype in the field experiment at two locations during 2016 and 2017 post rainy seasons (Chi-sq. probability < 0.001). Degree of freedom (d.f.).

Trait	Fixed term	Wald statistic	d.f	Wald/d.f.
DFL	G	2910.67	203	14.34
	Env	13258.52	7	1894.07
	G x Env	2995.49	1417	2.11
DM	G	1971.71	203	9.71
	Env	15023.16	7	2146.17
	G x Env	2159.1	1417	1.52
PLH	G	1550.15	203	7.64
	Env	1410.21	7	201.46
	G x Env	2128.73	1417	1.5
NB	G	10731.03	203	52.86
	Env	2862.51	7	408.93
	G x Env	2546.68	1383	1.84
NFW	G	13860.85	203	68.28
	Env	1691.8	7	241.69
	G x Env	2524.45	1383	1.83
NDW	G	3609.75	203	17.78
	Env	1385.25	7	197.89
	G x Env	2184.37	1383	1.58

**Table 2:** Trial means, for morphological traits and yield components of 204 genotype in the field experiment under two water treatments at two locations during 2016 and 2017 seasons.

Traits	Water		Range of predicted means		Heritability		
	Treatments	Trial mean	Minimum	Maximum	S.Ed	(h^2^)	SE
DFL	RF	60	53	65	1.293	0.45	0.107
	IRR	62	56	67	1.162	0.4	0.127
DM	RF	96	92	102	1.105	0.39	1.263
	IRR	98	95	102	0.883	0.32	0.485
PLH	RF	30	20.4	46.5	2.258	0.35	0.193
	IRR	38.5	26.4	54.8	2.353	0.34	1.228
NB	RF	1.767	0.475	5.957	0.572	0.37	0.051
	IRR	2.13	0.369	7.149	0.875	0.34	0.198
NFW	RF	1.829	0.441	6.291	0.57	0.38	0.084
	IRR	2.144	0.634	7.41	0.828	0.37	0.236
NDW	RF	0.297	0.078	1.044	0.096	0.31	0.013
	IRR	0.382	0.076	1.711	0.196	0.28	0.025
100SW	RF	22.31	9.62	46.57	2.187	0.71	0.117
	IRR	23.18	10.84	47.97	2.54	0.67	0.387
GY	RF	8.6	0.3333	38.03	1.997	0.27	0.11
	IRR	10.07	0.3667	61.3	2.683	0.33	0.501
BY	RF	22.17	0.9667	85.5	3.924	0.22	0.688
	IRR	27.19	1.967	147.8	5.66	0.29	0.468
HI	RF	0.41	0.235	0.592	0.058	0.19	0.008
	IRR	0.39	0.244	0.914	0.074	0.14	0.012
DTS	RF	6	4	8	0.488	0.26	0.076
	IRR	5	3	7	0.438	0.35	0.036

#### Variation in yield and yield components

The results of variance components (REML analysis) showed large variation (P < 0.001) among genotypes (G), environment (E) and the interaction (G x E) for yield and yield components under rainfed and irrigated conditions (Table 3). The mean of GY, BY and 100SW were higher under irrigated comparing to rainfed conditions, while the HI and the DTS were higher under rainfed condition (Table 3). The range of predicted mean for BY was broad under rainfed and irrigated conditions. Drought stress reduced the GY, BY and 100SW (14.9%, 18.5% and 3.8 respectively) relative to irrigated condition (Table 2). The high tolerant genotypes have higher seed yield and nodulation characteristics comparing to the susceptible genotypes under all environments (S2 Table). Heritability indices for GY and BY were higher under irrigation than in rainfed conditions, while the *h^2^* in 100SW was higher (0.71) under rainfed compared to irrigated conditions (0.67). The heritability for 100SW was higher than other morphological traits and yield components (Table 2).

**Table 3:** Components of variance of yield components and drought tolerance score of 204 genotype in the field experiment at two locations during 2016 and 2017 seasons (Chi-sq. probability < 0.001). Degree of freedom (d.f.).

Trait	Fixed term	Wald statistic	d.f	Wald/d.f.
100SW	G	7241.09	203	35.67
	Env	301.64	7	43.09
	G x Env	1764.42	1417	1.25
GY	G	2989.78	203	14.73
	Env	1256.21	7	179.46
	G x Env	2068.79	1417	1.46
BY	G	2239.99	203	11.03
	Env	3761.14	7	537.31
	G x Env	2044.03	1417	1.44
HI	G	602.79	203	2.97
	Env	280.37	7	40.05
	G x Env	1653.74	1417	1.17
DTS	G	1878.39	203	9.25
	Env	153.56	7	21.94
	G x Env	2280.44	1417	1.61

### Correlation between morphological and yield component traits

The phenotypic correlation coefficients among quantitative traits under rainfed and irrigated conditions are presented in Table 4. DFL had a significant positive correlation with other traits under drought and irrigated conditions and a negative correlation with 100SW under irrigated condition and with HI, and DTS under drought stress condition. Both DFL and DM correlated significantly with nodule observations, but these correlations were higher under drought stress than in irrigated conditions. A high significant and negative correlation was found between DTS and PLH, 100SW, GY, BY, NB, NDW and NFW under two water levels. PLH and 100SW were positively correlated with BY, DTS, NB, NDW and NFW under drought and irrigated conditions, whereas there was negative correlation between PLH and HI under drought stress condition only. There was no significant correlation between HI and 100SW under drought stress and with nodules characteristics under irrigated condition. BY showed high significant and positive correlation with GY (r = 0.83***, 0.82***), NB (r = 0.47***, 0.51***), NDW (r = 0.47***, 0.46***) and NFW (r = 0.49***, 0.53***) under drought and irrigated conditions, respectively (Table 4). A significant and negative correlation were found between HI and BY under drought and irrigated conditions and between HI and NB, NDW, NFW under drought condition. The GY per plant exhibited a significant positive correlation with HI (r = 0.26***, 0.28***), NB (r = 0.36***, 0.48***), NDW (r = 0.36***, 0.46***) and NFW (r = 0.38***, 0.52***) under drought and irrigated conditions, respectively. There was high and significant correlation between NB and NDW (r = 0.87***, 0.82***), NFW (r = 0.96***, 0.95***), also, the correlation was significant and positive between NDW and NFW (r = 0.89, 0.88) under drought and irrigated conditions, respectively (Table 4).

**Table 4:** Phenotypic correlation coefficients between different morphological and yield components in chickpea under drought and irrigated conditions.

Traits	DFL	DM	DRS	PLH	100SW	BY	GY	HI	NB	NDW	NFW
DFL	-	0.28***	0.053*	0.39***	-0.11***	0.34***	0.26***	-0.19***	0.25***	0.20***	0.27***
DM	0.53***	-	-0.033ns	0.39***	0.16***	0.50***	0.35***	-0.24***	0.37***	0.35***	0.40***
DRS	-0.12***	-0.11***	-	-0.21***	-0.31***	-0.17***	-0.23***	-0.16***	-0.14***	-0.13***	-0.15***
PLH	0.53***	0.47***	-0.33***	-	0.24***	0.59***	0.51***	-0.10***	0.46***	0.41***	0.48***
100SW	0.059*	0.19***	-0.22***	0.32***	-	0.28***	0.31***	0.058*	0.25***	0.22***	0.25***
BY	0.51***	0.53***	-0.28***	0.54***	0.33***	-	0.82***	-0.17***	0.51***	0.46***	0.53***
GY	0.41***	0.29***	-0.36***	0.47***	0.34***	0.83***	-	0.28***	0.48***	0.46***	0.52***
HI	-0.23***	-0.42***	-0.13***	-0.13***	0.02 ns	-0.22***	0.26***	-	-0.032ns	0.0038 ns	-0.024ns
NB	0.34***	0.48***	-0.18***	0.33***	0.35***	0.47***	0.36***	-0.18***	-	0.81***	0.95***
NDW	0.32***	0.47***	-0.19***	0.34***	0.31***	0.47***	0.36***	-0.17***	0.87***	-	0.88***
NFW	0.35***	0.49***	-0.21***	0.35***	0.35***	0.49***	0.38***	-0.18***	0.96***	0.89***	-

ns = not significant;

* = P<0.05;

** = P<0.0l;

*** = P<0.00l.

Upper diagonal represents the plants grown under irrigated condition and lower diagonal represents the plants grown under drought stress condition.

## Discussion

### Variation in morphological traits

Substantial phenotypic variation was found among genotypes by environments interaction for the different quantitative traits studied, indicating the presence of genotypic variability and different responses of genotypes to water deficit and possible selection of drought tolerant genotypes. This effect contributed to the largest variance component of the experiments [15]. Thus, the performance of some genotypes were largely in specific environments and genotypic differences were obtained from adaptive responses to the different environments as earlier reported by Anbessa and Bejiga [16], which observed differences among genotypes in their reactions to drought and 18 tolerant genotypes were identified based on drought response index (DRI).

The effectiveness of the imposed drought stress in rainfed plots was indicated by the differences of the means in the DFL, DM, PLH, NB, NDW and NFW between rainfed and irrigated regimes. The overall means of DFL and DM were less in rainfed compared to irrigated treatments (Table 2). The range of predicted means did not show this effect because some early duration genotypes flowered early in the drought treatment and a few late duration genotypes flowered later. These overall phenology differences were likely due to the required thermal time accumulation for flowering [17]. These results have indicated that, plants grown under rainfed conditions flowered and matured earlier than those under irrigated conditions and the same results were also reported by Saxena [18], and Kumar [5]. The extra earliness may be exploited in the improvement of chickpea for short growing environments, as the flowering and pods setting of the crop occur before water stress becomes a serious limiting factor [16]. The present study has shown that the level of drought has a major impact on the production and abortion of pods and hence on seed yield, therefor selected early flowering and maturity genotypes help the plants to avoid and escape from water deficit in crucial stage. The mean of plant height was greater under irrigated conditions in compared to rainfed treatment (Table 2). In fact, water deficit at the generative stage decreases the plants height. These results are in line with that reported by Shamsi [19], Hajibabaee [20] and Maqbool [21].

The variability in symbiotic efficiency of various strains provides an impression that, the N_2_-fixing ability of symbiotic bacteria could be improved by strain selection. In this study, the seeds were inoculated with *Mesorhizobium cicer* CP-36, CP39 strains, these rhizobia have been previously evaluated, that indicating significant variation in the symbiotic performance referring “probably” to the differences in the rhizobia symbiotic efficiency and the degree of compatibility with the host plant [7, 22]. Our results indicated that the drought stress had affected all nodules observations by suppressing the growth of the nodules, these indices have been used to estimate genotypes with high nodulation and production (Table 2). The high sensitivity of chickpea nodule development as compared to other plant parts suggests that drought stress specifically affected nodule development. Inhibition of nodule development in the stressed plants may due to restriction of carbohydrate transport from leaves to nodule [23]. The tolerant genotypes have a complex mechanism for maintaining cell turgor and accumulation of proline as a consequence of the reduction in the osmotic adjustment under drought stress. The effect of drought on nodule formations was clear in this experiment to this reason, study the interaction between them are important for drought tolerance research in the future. The highest heritability (*h^2^*) values were for all morphological traits were observed under the drought stress environment whereas it turned to be less when irrigated (Table 2), these results were validated by Krishnamurthy *et al*. [24]. The nodulation pattern for genotypes in this study is in agreements with the results of other studies in nodules biomass and weight reported by Kyei-Boahen *et al*. [22]; Pimratch *et al*. [25]; Esfahani *et al*. [7].

### Variation in yield and yield components

The high significance value (*P* < 0.001) for all attributes showed considerable variation for these traits among chickpea genotypes, environments, and their interaction in response to water deficit (Table 3) [16, 26, 27]. From these results, drought stress causes a significant reduction in yield and yield components (Table 2), by affecting both plant growth and growth period, these findings are in line with that reported by Singh [28]; Dogan *et al.,* [29]. Similarly, Turner, [30]; Leport *et al.,* [31] and Yaqoob *et al*., [32] reported that, drought stress decreased growth development and grain yield in chickpea, Karadavut *et al*., [33] in faba bean and Hajibabaee *et al*., [20] in Maize. This suggested that it may be more relevant to focus on breeding for drought tolerance under multi-environments and traits such as nodule biomass, yield and yield components. Minimal decrease in yield and nodule biomass was found in the most tolerant genotypes such as IG70399, IG8256, IG71832, IG70270 and IG70272 (S1 Table). These genotypes represent an ideal material for further characterization of underlying mechanisms of tolerance involved and are expected to have much wider adaptability as were these selected not simply on the basis of seed yield but also by DTS as a result we can use it as a source for crossing programs. When subjected to water stress, both total biomass and seed yield decreased to a greater extent the earlier the stress was imposed. However, the seed yield decreased more than the biomass with the stress treatments, so the harvest index also decreased linearly with the duration of water stress [31].

The heritability indices were not only high for the phenological traits, but also for the 100SW and grain yield in this environment indicating the possibilities of a direct selection for yield in chickpea. Canci and Toker [27] reported that seed weight had high heritability across changing environmental conditions, and it should be used for selection in early breeding generations. However, a higher confidence level can be placed on this heritability index as this is likely to be reproducible across environments [24].

### Correlation coefficient analysis

The correlation coefficient analysis showed a positive significant correlation between DFL and DM, PLH and GY. Similar results were reported by Rao and Kumar [34]; Patil *et al.,* [35] for plant height, day to maturity and Yucel *et al.,* [36]; Orange *et al.,* [37] for grain yield, whereas a contrast result was reported by Yucel *et al.,* [36] for the negative correlation between DFL and PLH. In this case, it could be suitable to select short bloom lines for increasing GY per plant.

The correlation values for DFL and DM with nodule observations were higher under drought stress than in irrigated conditions, which indicates the response of these genotypes under drought stress condition (Table 4). The results showed early maturity genotypes with high nodulation have higher production and are recommended for planting in the rainfed environments. The significant positive correlation between PLH and 100SW, BY and GY indicated that cultivars with higher PLH contribute more to GY [35, 36]. However, there was a negative correlation between PLH and HI. Anlarsal *et al.,* [38] indicated that an increase in PLH leads to a decrease in HI. 100SW showed a positive significant correlation with GY, NB, NDW and NFW.

In this study, the results indicated that plants with higher nodule biomass, and higher number of pods per plant have higher grain yield. These traits could be used effectively for screening high yielding genotypes under drought stress conditions. Similar results were also reported by Patil *et al*., [35] for GY and Bhuiyan *et al.,* [39] for NB and NDW. HI showed a positive correlation with GY and a negative correlation with BY. Therefore, this result indicated that HI might serve to identify chickpea genotypes with higher GY per plant [36]. The GY per plant exhibited a significant positive correlation with BY, NB, NDW and NFW. The results from the current study suggest that high nodulation and production genotypes can recommended for the farmer to avoid added huge quantities of fertilizer and to save money, which were in accordance with those reported by Bhuiyan *et al*., [39] for NDW and Kyei-Boahen *et al.,* [22] for NB and NDW. On the contrary Bhuiyan *et al.,* [39] reported, there was no significant correlations between grain yield and nodule dry weight. These results are in line with what was suggested by Guler *et al.,* [40], that, any positive increase in such traits accelerates the boost in GY per plant. The main concerns of breeders are to achieve an increase in chickpea yield. Yield and its components are multigenic traits, which are strongly influenced by the environment and other factors both known and yet to be identified. To this end, emphasis should be given to the development of chickpea genotypes with high growth rate, and nodulations to improve grain yield.

Regulation of stomatal density and distribution in *Arabidopsis thaliana* [41], identification of disease resistance subtilizes target substrate and in the elucidation of their participation in the immune priming activation [42], identification of differentially expressed in response to drought induced by PEG 6000 in *Populus canadensis* leaves [43] and Responses to abiotic stress such as drought and salt stress in desert tree *Prosopis juliflora* [44].

### Conclusion

Drought stress signaling is an important area with respect to an increase in plant productivity. Drought is a worldwide problem, constraining global crop production and quality seriously, and recent global climate change has made this situation more serious. This work permitted to purpose of several indices to predict relative tolerance to drought with high N- fixation through stable nodulation for tolerant chickpea genotypes. The results showed significant variation between genotypes, environments and the interaction for morphological, yield and yield components. Drought stress reduced these traits and a higher reduction was noticed in nodules characteristics, biological and grain yield. The tolerant genotypes have higher seed yield and nodulation characteristics compared to susceptible genotypes under all environments.

## Supporting information

S1 Table Passport information of the 204 chickpea germplasm used in this study. (DOCX)

S2 Table Agronomic traits under water treatments. Mean value for 204 chickpea germplasm under two water treatments, two locations and two years. DTS = drought tolerance, GY = grain yield (g/plant), NB = nodule biomass (m^3^), NFW = nodule fresh weight (g/plant), NDW = nodule dry weight (g/plant).
(DOCX)
