# Supplementary material for: The interaction between drought stress and nodule formation under multiple environments in chickpea
Source: PLoS One. 2022 Oct 27;17(10):e0276732. doi: 10.1371/journal.pone.0276732 (PMC9612560; doi:10.1371/journal.pone.0276732)
Supplement: S1 Table — (DOCX) [file pone.0276732.s001.docx]

Table1 supplement. Names and origin for 204 chickpea germplasm.

| **IG** | **Taxa** | **Germplasm** | **Ori** | **Province** | **LON** | **LAT** |
| --- | --- | --- | --- | --- | --- | --- |
| IG6035 | Cicer arietinum | accessions | RUS | Saratov | E 46 02 | N 51 32 |
| IG6041 | Cicer arietinum | accessions | SDN | Al Wusta | E33 30 | N14 24 |
| IG6050 | Cicer arietinum | accessions | IND | Punjab | E75 10 | N30 49 |
| IG6057 | Cicer arietinum | accessions | PAK | NWF | E71 40 | N34 01 |
| IG6058 | Cicer arietinum | accessions | PAK | Punjab | E72 26 | N33 46 |
| IG6109 | Cicer arietinum | accessions | IND | Bihar | E85 56 | N26 00 |
| IG6111 | Cicer arietinum | accessions | IND | Punjab | E76 46 | N30 44 |
| IG6113 | Cicer arietinum | accessions | IND | Uttar Pradesh | E80 19 | N26 28 |
| IG6446 | Cicer arietinum | accessions | IND | Delhi | E77 13 | N28 39 |
| IG6468 | Cicer arietinum | accessions | TUN | Nabul | E10 59 | N36 47 |
| IG7714 | Cicer arietinum | accessions | IND | Maharashtra | E75 00 | N19 30 |
| IG7717 | Cicer arietinum | accessions | IND | Punjab | E75 51 | N30 56 |
| IG7722 | Cicer arietinum | accessions | IND | Madhya Pradesh | E77 22 | N23 14 |
| IG7758 | Cicer arietinum | accessions | IND | Uttar Pradesh | E80 55 | N26 51 |
| IG8256 | Cicer arietinum | accessions | IND | Punjab | E74 45 | N30 41 |
| IG8447 | Cicer arietinum | accessions | SDN | Al Wusta | E33 30 | N14 24 |
| IG8914 | Cicer arietinum | accessions | PAK | Punjab | E74 23 | N31 39 |
| IG8985 | Cicer arietinum | accessions | IND | Rajasthan | E76 39 | N26 18 |
| IG9088 | Cicer arietinum | accessions | IND | Rajasthan | E72 27 | N24 48 |
| IG9239 | Cicer arietinum | accessions | ITA | Toscana | E11 11 | N43 45 |
| IG9244 | Cicer arietinum | accessions | ITA | Toscana | E11 11 | N43 45 |
| IG9413 | Cicer arietinum | accessions | IND | Madhya Pradesh | E75 04 | N24 04 |
| IG9425 | Cicer arietinum | accessions | PAK | Punjab | E72 22 | N31 19 |
| IG9430 | Cicer arietinum | accessions | IND | Himachal Pradesh | E76 16 | N31 29 |
| IG9431 | Cicer arietinum | accessions | IND | Himachal Pradesh | E76 16 | N31 29 |
| IG9433 | Cicer arietinum | accessions | IND | Punjab | E75.67 | N30.82 |
| IG9434 | Cicer arietinum | accessions | IND | Punjab | E75.53 | N30.06 |
| IG9435 | Cicer arietinum | accessions | IND | Punjab | E75.84 | N30.24 |
| IG9436 | Cicer arietinum | accessions | IND | Punjab | E76 25 30 | N30 17 20 |
| IG9437 | Cicer arietinum | accessions | IND | Punjab | E74.75 | N30.66 |
| IG9438 | Cicer arietinum | accessions | IND | Punjab | E74.75 | N30.66 |
| IG9439 | Cicer arietinum | accessions | IND | Punjab | E75.40 | N32.04 |
| IG9440 | Cicer arietinum | accessions | IND | Punjab | E75.40 | N32.04 |
| IG9441 | Cicer arietinum | accessions | IND | Punjab | E75.91 | N31.53 |
| IG9442 | Cicer arietinum | accessions | IND | Himachal Pradesh | E76 17 | N31 29 |
| IG9584 | Cicer arietinum | accessions | NPL | Narayani | E84 59 | N27 10 |
| IG9586 | Cicer arietinum | accessions | IND | Punjab | E75 24 | N32 03 |
| IG9627 | Cicer arietinum | accessions | IND | Punjab | E75.91 | N31.45 |
| IG9629 | Cicer arietinum | accessions | IND | Himachal Pradesh | E76 16 | N31 29 |
| IG9630 | Cicer arietinum | accessions | IND | Himachal Pradesh | E76 44 02 | N31 21 59 |
| IG69605 | Cicer arietinum | accessions | TUN | Bajah | E 09 11 | N 36 43 |
| IG69620 | Cicer arietinum | accessions | IND | Haryana | E75 42 | N29 10 |
| IG70246 | Cicer arietinum | accessions | PAK | Sindh | E67 32 | N24 44 |
| IG70248 | Cicer arietinum | accessions | PAK | Sindh | E67 52 | N24 33 |
| IG70249 | Cicer arietinum | accessions | PAK | Sindh | E68 05 | N24 35 |
| IG70252 | Cicer arietinum | accessions | PAK | Sindh | E68 51 | N24 53 |
| IG70253 | Cicer arietinum | accessions | PAK | Sindh | E68 44 | N24 57 |
| IG70255 | Cicer arietinum | accessions | PAK | Sindh | E68 25 | N25 25 |
| IG70262 | Cicer arietinum | accessions | PAK | Sindh | E68 45 | N25 28 |
| IG70265 | Cicer arietinum | accessions | PAK | Sindh | E69 01 | N25 32 |
| IG70269 | Cicer arietinum | accessions | PAK | Sindh | E69 25 | N25 50 |
| IG70270 | Cicer arietinum | accessions | PAK | Sindh | E68 51 | N26 02 |
| IG70272 | Cicer arietinum | accessions | PAK | Sindh | E68 41 | N25 55 |
| IG70273 | Cicer arietinum | accessions | PAK | Sindh | E68 35 | N25 53 |
| IG70275 | Cicer arietinum | accessions | PAK | Sindh | E68 29 | N25 44 |
| IG70277 | Cicer arietinum | accessions | PAK | Sindh | E68 25 | N25 49 |
| IG70278 | Cicer arietinum | accessions | PAK | Sindh | E68 18 | N26 00 |
| IG70280 | Cicer arietinum | accessions | PAK | Sindh | E68 16 | N26 08 |
| IG70285 | Cicer arietinum | accessions | PAK | Sindh | E68 24 | N26 11 |
| IG70286 | Cicer arietinum | accessions | PAK | Sindh | E68 25 | N26 15 |
| IG70290 | Cicer arietinum | accessions | PAK | Sindh | E67 56 | N26 19 |
| IG70291 | Cicer arietinum | accessions | PAK | Sindh | E67 58 | N26 30 |
| IG70293 | Cicer arietinum | accessions | PAK | Sindh | E67 56 | N26 36 |
| IG70294 | Cicer arietinum | accessions | PAK | Sindh | E67 58 | N26 38 |
| IG70297 | Cicer arietinum | accessions | PAK | Sindh | E67 51 | N26 41 |
| IG70299 | Cicer arietinum | accessions | PAK | Sindh | E67 45 | N26 43 |
| IG70304 | Cicer arietinum | accessions | PAK | Sindh | E67 40 | N26 27 |
| IG70305 | Cicer arietinum | accessions | PAK | Sindh | E67 50 | N26 25 |
| IG70308 | Cicer arietinum | accessions | PAK | Sindh | E68 01 | N26 40 |
| IG70309 | Cicer arietinum | accessions | PAK | Sindh | E67 40 | N27 01 |
| IG70311 | Cicer arietinum | accessions | PAK | Sindh | E67 51 | N27 04 |
| IG70312 | Cicer arietinum | accessions | PAK | Sindh | E68 00 | N27 09 |
| IG70313 | Cicer arietinum | accessions | PAK | Sindh | E68 03 | N27 18 |
| IG70316 | Cicer arietinum | accessions | PAK | Sindh | E68 13 | N27 34 |
| IG70328 | Cicer arietinum | accessions | PAK | Sindh | E68 21 | N27 40 |
| IG70330 | Cicer arietinum | accessions | PAK | Sindh | E68 24 | N27 49 |
| IG70332 | Cicer arietinum | accessions | PAK | Sindh | E68 30 | N27 49 |
| IG70334 | Cicer arietinum | accessions | PAK | Sindh | E68 38 | N27 54 |
| IG70335 | Cicer arietinum | accessions | PAK | Sindh | E68 39 | N27 57 |
| IG70336 | Cicer arietinum | accessions | PAK | Sindh | E68 28 | N28 15 |
| IG70338 | Cicer arietinum | accessions | PAK | Sindh | E68 41 | N27 49 |
| IG70340 | Cicer arietinum | accessions | PAK | Sindh | E68 49 | N27 46 |
| IG70342 | Cicer arietinum | accessions | PAK | Sindh | E68 04 | N27 42 |
| IG70345 | Cicer arietinum | accessions | PAK | Sindh | E69 06 | N27 48 |
| IG70346 | Cicer arietinum | accessions | PAK | Sindh | E69 13 | N27 58 |
| IG70347 | Cicer arietinum | accessions | PAK | Sindh | E69 28 | N28 04 |
| IG70348 | Cicer arietinum | accessions | PAK | Sindh | E69 44 | N28 04 |
| IG70349 | Cicer arietinum | accessions | PAK | Sindh | E69 39 | N28 05 |
| IG70350 | Cicer arietinum | accessions | PAK | Sindh | E69 41 | N28 11 |
| IG70351 | Cicer arietinum | accessions | PAK | Punjab | E69 59 | N28 13 |
| IG70352 | Cicer arietinum | accessions | PAK | Punjab | E70 19 | N28 26 |
| IG70355 | Cicer arietinum | accessions | PAK | Punjab | E70 39 | N28 38 |
| IG70357 | Cicer arietinum | accessions | PAK | Punjab | E70 44 | N28 56 |
| IG70359 | Cicer arietinum | accessions | PAK | Punjab | E70 59 | N29 12 |
| IG70361 | Cicer arietinum | accessions | PAK | Punjab | E70 54 | N29 40 |
| IG70363 | Cicer arietinum | accessions | PAK | Punjab | E71 29 | N30 11 |
| IG70369 | Cicer arietinum | accessions | PAK | Punjab | E71 18 | N30 40 |
| IG70370 | Cicer arietinum | accessions | PAK | Punjab | E71 33 | N30 32 |
| IG70371 | Cicer arietinum | accessions | PAK | Punjab | E71 14 | N30 34 |
| IG70374 | Cicer arietinum | accessions | PAK | Punjab | E71 13 | N31 15 |
| IG70375 | Cicer arietinum | accessions | PAK | Punjab | E71 13 | N31 21 |
| IG70377 | Cicer arietinum | accessions | PAK | Punjab | E71 23 | N31 27 |
| IG70379 | Cicer arietinum | accessions | PAK | Punjab | E71 23 | N31 25 |
| IG70381 | Cicer arietinum | accessions | PAK | Punjab | E71 26 | N31 16 |
| IG70383 | Cicer arietinum | accessions | PAK | Punjab | E71 26 | N31 13 |
| IG70384 | Cicer arietinum | accessions | PAK | Punjab | E71 28 | N31 07 |
| IG70388 | Cicer arietinum | accessions | PAK | Punjab | E71 28 | N31 02 |
| IG70389 | Cicer arietinum | accessions | PAK | Punjab | E71 38 | N31 23 |
| IG70390 | Cicer arietinum | accessions | PAK | Punjab | E71 54 | N31 53 |
| IG70393 | Cicer arietinum | accessions | PAK | Punjab | E71 50 | N31 23 |
| IG70394 | Cicer arietinum | accessions | PAK | Punjab | E71 49 | N31 32 |
| IG70398 | Cicer arietinum | accessions | PAK | Punjab | E71 42 | N31 38 |
| IG70399 | Cicer arietinum | accessions | PAK | Punjab | E71 31 | N31 38 |
| IG70401 | Cicer arietinum | accessions | PAK | Punjab | E71 31 | N31 41 |
| IG70402 | Cicer arietinum | accessions | PAK | Punjab | E71 39 | N31 47 |
| IG70408 | Cicer arietinum | accessions | PAK | Punjab | E71 29 | N31 30 |
| IG70409 | Cicer arietinum | accessions | PAK | Punjab | E71 44 | N31 48 |
| IG70410 | Cicer arietinum | accessions | PAK | Punjab | E71 51 | N31 50 |
| IG70413 | Cicer arietinum | accessions | PAK | Punjab | E71 55 | N31 46 |
| IG70416 | Cicer arietinum | accessions | PAK | Punjab | E71 54 | N31 53 |
| IG70419 | Cicer arietinum | accessions | PAK | Punjab | E71 50 | N32 01 |
| IG70421 | Cicer arietinum | accessions | PAK | Punjab | E71 32 | N32 35 |
| IG70427 | Cicer arietinum | accessions | PAK | Punjab | E71 46 | N32 41 |
| IG70428 | Cicer arietinum | accessions | PAK | Punjab | E72 28 | N33 04 |
| IG70429 | Cicer arietinum | accessions | PAK | Punjab | E72 32 | N33 32 |
| IG70430 | Cicer arietinum | accessions | PAK | Punjab | E72 28 | N33 21 |
| IG70432 | Cicer arietinum | accessions | PAK | Punjab | E72 28 | N33 10 |
| IG70434 | Cicer arietinum | accessions | PAK | Punjab | E72 25 | N32 58 |
| IG70556 | Cicer arietinum | accessions | RUS | Volgograd | E 45 24 | N 50 06 |
| IG70755 | Cicer arietinum | accessions | PAK | Sindh | E68 50 | N24 40 |
| IG70760 | Cicer arietinum | accessions | PAK | Sindh | E68 26 | N26 15 |
| IG70761 | Cicer arietinum | accessions | PAK | Sindh | E68 00 | N26 40 |
| IG70762 | Cicer arietinum | accessions | PAK | Sindh | E68 11 | N26 51 |
| IG70763 | Cicer arietinum | accessions | PAK | Sindh | E68 54 | N27 42 |
| IG70764 | Cicer arietinum | accessions | PAK | Sindh | E68 42 | N27 58 |
| IG70766 | Cicer arietinum | accessions | PAK | Sindh | E68 30 | N28 16 |
| IG70767 | Cicer arietinum | accessions | PAK | Sindh | E68 30 | N27 55 |
| IG70768 | Cicer arietinum | accessions | PAK | Sindh | E68 18 | N27 32 |
| IG70770 | Cicer arietinum | accessions | PAK | Punjab | E70 20 | N28 22 |
| IG70772 | Cicer arietinum | accessions | PAK | Punjab | E73 31 | N30 49 |
| IG70773 | Cicer arietinum | accessions | PAK | Punjab | E72 50 | N29 22 |
| IG70774 | Cicer arietinum | accessions | PAK | Punjab | E71 15 | N30 04 |
| IG70775 | Cicer arietinum | accessions | PAK | Punjab | E71 13 | N31 12 |
| IG70776 | Cicer arietinum | accessions | PAK | Punjab | E71 08 | N31 40 |
| IG70777 | Cicer arietinum | accessions | PAK | Punjab | E72 58 | N31 04 |
| IG70778 | Cicer arietinum | accessions | PAK | Punjab | E72 43 | N31 10 |
| IG70779 | Cicer arietinum | accessions | PAK | Punjab | E73 25 | N31 20 |
| IG70780 | Cicer arietinum | accessions | PAK | Punjab | E74 06 | N31 27 |
| IG70781 | Cicer arietinum | accessions | PAK | Sindh | E68 42 | N27 58 |
| IG70782 | Cicer arietinum | accessions | PAK | Punjab | E74 21 | N32 23 |
| IG70783 | Cicer arietinum | accessions | PAK | Punjab | E74 06 | N32 35 |
| IG70784 | Cicer arietinum | accessions | PAK | Punjab | E74 01 | N32 41 |
| IG70785 | Cicer arietinum | accessions | PAK | Punjab | E72 29 | N32 55 |
| IG70787 | Cicer arietinum | accessions | PAK | Punjab | E72 06 | N33 30 |
| IG70788 | Cicer arietinum | accessions | PAK | NWF | E71 54 | N34 30 |
| IG70789 | Cicer arietinum | accessions | PAK | Sindh | E70 13 | N25 19 |
| IG70822 | Cicer arietinum | accessions | PAK | Punjab | E73 09 | N31 25 |
| IG71832 | Cicer arietinum | accessions | TUR | Antakya | E36 10 22 | N36 34 14 |
| IG73369 | Cicer arietinum | accessions | IND | Madhya Pradesh | E78 27 | N25 41 |
| IG73381 | Cicer arietinum | accessions | IND | Rajasthan | E73 52 | N29 56 |
| IG73382 | Cicer arietinum | accessions | IND | Rajasthan | E73 52 | N29 56 |
| IG73386 | Cicer arietinum | accessions | IND | Madhya Pradesh | E81 18 | N24 32 |
| IG73388 | Cicer arietinum | accessions | TUR | Antakya | E35 55 | N36 21 |
| IG73390 | Cicer arietinum | accessions | TUR | Antakya | E36 16 | N36 22 |
| IG73394 | Cicer arietinum | accessions | TUR | Antakya | E35 57 | N36 06 |
| IG74021 | Cicer arietinum | accessions | IND | Bihar | E85 41 | N25 59 |
| IG74929 | Cicer arietinum | accessions | PAK | Punjab | E74 20 | N31 33 |
| IG74940 | Cicer arietinum | accessions | IND | Punjab | E76.71 | N31.04 |
| IG74979 | Cicer arietinum | accessions | NPL | Seti | E80.78 | N28.56 |
| IG74994 | Cicer arietinum | accessions | IND | Delhi | E77 14 | N28 39 |
| IG74995 | Cicer arietinum | accessions | IND | Jammu & Kashmir | E74.85 | N32.71 |
| IG75029 | Cicer arietinum | accessions | IND | Haryana | E76.16 | N29.47 |
| IG75360 | Cicer arietinum | accessions | IND | Rajasthan | E75 48 | N26 56 |
| IG75406 | Cicer arietinum | accessions | IND | Jammu & Kashmir | E74.85 | N32.71 |
| IG114795 | Cicer arietinum | accessions | ETH | Shewa | E40 10 | N09 01 |
| IG115380 | Cicer arietinum | accessions | NPL | Mahakali | E80 22 13 | N28 40 36 |
| IG115390 | Cicer arietinum | accessions | NPL | Mahakali | E80 21 35 | N28 50 00 |
| IG117696 | Cicer arietinum | accessions | PAK | Punjab | E71 30 26 | N32 10 53 |
| IG117697 | Cicer arietinum | accessions | PAK | Punjab | E71 29 38 | N32 04 53 |
| IG117698 | Cicer arietinum | accessions | PAK | Punjab | E71 29 00 | N32 02 05 |
| IG117699 | Cicer arietinum | accessions | PAK | Punjab | E71 27 24 | N31 56 15 |
| IG117700 | Cicer arietinum | accessions | PAK | Punjab | E71 03 29 | N31 35 31 |
| IG117703 | Cicer arietinum | accessions | PAK | Punjab | E72 26 29 | N29 40 38 |
| IG117708 | Cicer arietinum | accessions | PAK | Punjab | E72 34 36 | N29 39 10 |
| IG117714 | Cicer arietinum | accessions | PAK | Punjab | E72 39 49 | N29 41 20 |
| IG117718 | Cicer arietinum | accessions | PAK | Punjab | E72 01 03 | N29 29 46 |
| IG117728 | Cicer arietinum | accessions | PAK | Punjab | E72 51 00 | N29 24 52 |
| IG125018 | Cicer arietinum | accessions | AZE | Lankaran | E 48 50 | N 38 45 |
| IG125021 | Cicer arietinum | accessions | RUS | Saratov | E46 47 | N50 57 |
| IG125028 | Cicer arietinum | accessions | RUS | Volgograd | E44 29 | N48 42 |
| IG128430 | Cicer arietinum | accessions | AZE | Lankaran | E 48 47 | N 38 46 |
| IG128434 | Cicer arietinum | accessions | AZE | Lenkoran' | E 48 50 | N 38 44 |
| IG128465 | Cicer arietinum | accessions | ITA | Lazio | E 12 39 | N 41 53 |
| IG128506 | Cicer arietinum | accessions | AZE | Lankaran | E48 49 | N38 45 |
| IG131983 | Cicer arietinum | accessions | EGY | Qena | E 32 38 | N 25 41 |
| IG131985 | Cicer arietinum | accessions | EGY | Qena | E 32 42 | N 25 44 |
| IG132032 | Cicer arietinum | accessions | SYR | Lattakia | E35 48 | N35 32 |
| IG134532 | Cicer arietinum | accessions | IND | Bihar | E085 41 | N25 59 |
| IG134564 | Cicer arietinum | accessions | PAK | North-West Frontier | E 71 32 | N 34 00 |
| Genesis090 | Cicer arietinum | breeding line | AUS | New south wales | E00541 | N 52 1859 |
| pbahattrick | Cicer arietinum | breeding line | AUS | New south wales | E00541 | N 52 1859 |
| pbapistol | Cicer arietinum | breeding line | AUS | New south wales | E00541 | N 52 1859 |
| howzat | Cicer arietinum | breeding line | AUS | New south wales | E00541 | N 52 1859 |
| Pbabaumary | Cicer arietinum | breeding line | AUS | New south wales | E00541 | N 52 1859 |
