# Supplementary material for: The interaction between drought stress and nodule formation under multiple environments in chickpea
Source: PLoS One. 2022 Oct 27;17(10):e0276732. doi: 10.1371/journal.pone.0276732 (PMC9612560; doi:10.1371/journal.pone.0276732)
Supplement: S2 Table — Mean value for 204 chickpea germplasm under two water treatments, two locations and two years. DTS = drought tolerance, GY = grain yield (g/plant), NB = nodule biomass (m3), NFW = nodule fresh weight (g/plant), NDW = nodule dry weight (g/plant). (DOCX) [file pone.0276732.s002.docx]

**Table S2.** Mean value for 204 chickpea germplasm under two water treatments, two locations and two years. DTS= drought tolerance, GY= grain yield (g/plant), NB= nodule biomass (m³), NFW= nodule fresh weight (g/plant), NDW= nodule dry weight (g/plant).

| S NO. | Genotype | DR | GY | NB | NDW | NFW |
| --- | --- | --- | --- | --- | --- | --- |
| 1 | IG114795 | 4.2 | 9.79 | 5.13 | 0.60 | 4.56 |
| 2 | IG132032 | 4.4 | 11.65 | 4.11 | 0.69 | 4.15 |
| 3 | IG70270 | 4.5 | 14.05 | 4.98 | 1.05 | 5.27 |
| 4 | IG70272 | 4.5 | 13.56 | 5.11 | 0.53 | 5.44 |
| 5 | IG70399 | 3.8 | 21.91 | 3.57 | 0.54 | 3.92 |
| 6 | IG117703 | 3.6 | 13.20 | 2.69 | 0.48 | 2.92 |
| 7 | IG71832 | 4.0 | 14.39 | 5.96 | 1.04 | 6.33 |
| 8 | IG73394 | 4.3 | 12.96 | 5.11 | 0.71 | 5.43 |
| 9 | IG8256 | 4.2 | 17.36 | 3.33 | 0.55 | 3.16 |
| 10 | Genesis090 | 4.0 | 12.70 | 3.65 | 0.62 | 3.71 |
| 11 | IG117698 | 4.3 | 10.54 | 2.32 | 0.33 | 2.51 |
| 12 | IG117703 | 5.7 | 9.25 | 2.61 | 0.37 | 2.70 |
| 13 | IG117728 | 5.4 | 7.45 | 2.11 | 0.28 | 2.22 |
| 14 | IG128465 | 5.5 | 9.72 | 2.40 | 0.45 | 2.58 |
| 15 | IG134532 | 6.3 | 10.74 | 2.69 | 0.43 | 2.74 |
| 16 | IG6035 | 5.5 | 6.00 | 1.95 | 0.43 | 2.03 |
| 17 | IG6058 | 4.7 | 10.46 | 2.21 | 0.43 | 2.45 |
| 18 | IG6109 | 6.2 | 5.30 | 3.46 | 0.41 | 2.35 |
| 19 | IG6113 | 5.0 | 9.15 | 2.08 | 0.34 | 2.17 |
| 20 | IG70246 | 6.4 | 7.61 | 2.56 | 0.42 | 2.81 |
| 21 | IG70248 | 5.3 | 6.62 | 2.11 | 0.34 | 2.19 |
| 22 | IG70249 | 5.5 | 10.13 | 3.10 | 0.54 | 3.38 |
| 23 | IG70252 | 5.8 | 9.82 | 2.76 | 0.42 | 2.88 |
| 24 | IG70253 | 4.8 | 9.56 | 1.66 | 0.33 | 1.87 |
| 25 | IG70255 | 5.3 | 10.71 | 2.17 | 0.40 | 2.38 |
| 26 | IG70262 | 5.6 | 9.84 | 2.10 | 0.34 | 2.15 |
| 27 | IG70265 | 5.0 | 9.50 | 2.19 | 0.40 | 2.52 |
| 28 | IG70269 | 5.6 | 6.50 | 1.97 | 0.37 | 2.24 |
| 29 | IG70273 | 5.2 | 7.17 | 2.03 | 0.34 | 2.13 |
| 30 | IG70278 | 5.7 | 6.55 | 2.08 | 0.34 | 2.06 |
| 31 | IG70285 | 5.7 | 8.35 | 2.42 | 0.28 | 2.48 |
| 32 | IG70290 | 4.7 | 8.40 | 2.24 | 0.42 | 2.46 |
| 33 | IG70293 | 5.3 | 7.75 | 1.83 | 0.29 | 1.85 |
| 34 | IG70304 | 5.3 | 9.38 | 2.19 | 0.37 | 2.37 |
| 35 | IG70313 | 5.4 | 8.02 | 2.69 | 0.42 | 2.77 |
| 36 | IG70316 | 5.6 | 7.40 | 2.47 | 0.37 | 2.46 |
| 37 | IG70332 | 5.1 | 12.00 | 2.64 | 0.43 | 2.51 |
| 38 | IG70335 | 5.8 | 5.34 | 2.37 | 0.33 | 2.38 |
| 39 | IG70336 | 5.3 | 7.41 | 3.66 | 0.40 | 2.51 |
| 40 | IG70338 | 5.2 | 8.64 | 2.83 | 0.44 | 2.94 |
| 41 | IG70340 | 5.4 | 6.17 | 2.05 | 0.40 | 2.16 |
| 42 | IG70342 | 5.7 | 6.57 | 3.34 | 0.46 | 3.48 |
| 43 | IG70350 | 6.0 | 6.16 | 2.45 | 0.36 | 2.74 |
| 44 | IG70352 | 5.7 | 8.82 | 2.50 | 0.43 | 2.67 |
| 45 | IG70355 | 6.3 | 9.25 | 2.49 | 0.31 | 2.42 |
| 46 | IG70359 | 4.5 | 12.09 | 1.90 | 0.29 | 1.79 |
| 47 | IG70371 | 4.5 | 8.23 | 1.63 | 0.34 | 1.77 |
| 48 | IG70379 | 4.3 | 9.35 | 1.85 | 0.37 | 1.98 |
| 49 | IG70384 | 5.0 | 10.11 | 2.19 | 0.31 | 2.23 |
| 50 | IG70393 | 5.7 | 8.70 | 2.79 | 0.52 | 2.90 |
| 51 | IG70394 | 5.0 | 7.82 | 1.78 | 0.35 | 1.88 |
| 52 | IG70398 | 4.4 | 8.43 | 1.87 | 0.37 | 2.05 |
| 53 | IG70427 | 4.5 | 8.82 | 1.74 | 0.31 | 1.79 |
| 54 | IG70428 | 5.3 | 8.05 | 1.65 | 0.32 | 1.70 |
| 55 | IG70429 | 4.9 | 10.31 | 2.52 | 0.44 | 2.59 |
| 56 | IG70430 | 5.5 | 9.05 | 2.41 | 0.43 | 2.39 |
| 57 | IG70432 | 5.2 | 9.49 | 2.15 | 0.43 | 2.31 |
| 58 | IG70434 | 5.5 | 9.36 | 2.31 | 0.45 | 2.40 |
| 59 | IG70556 | 6.1 | 5.46 | 1.89 | 0.33 | 2.04 |
| 60 | IG70755 | 4.5 | 7.59 | 2.06 | 0.33 | 2.22 |
| 61 | IG70763 | 5.5 | 8.34 | 2.11 | 0.34 | 2.14 |
| 62 | IG70764 | 5.6 | 5.93 | 1.86 | 0.31 | 1.89 |
| 63 | IG70766 | 5.7 | 7.62 | 2.64 | 0.41 | 2.77 |
| 64 | IG70767 | 4.9 | 11.24 | 3.05 | 0.46 | 2.81 |
| 65 | IG70770 | 6.0 | 9.32 | 2.01 | 0.37 | 2.11 |
| 66 | IG70772 | 4.9 | 7.74 | 2.08 | 0.30 | 2.04 |
| 67 | IG70776 | 4.4 | 8.49 | 2.43 | 0.38 | 2.58 |
| 68 | IG70777 | 6.0 | 8.52 | 1.63 | 0.53 | 1.78 |
| 69 | IG70779 | 4.7 | 10.44 | 1.86 | 0.32 | 1.85 |
| 70 | IG70781 | 5.3 | 7.59 | 1.69 | 0.37 | 1.76 |
| 71 | IG70782 | 5.5 | 6.64 | 1.78 | 0.37 | 1.90 |
| 72 | IG70785 | 5.7 | 10.03 | 2.22 | 0.41 | 2.29 |
| 73 | IG73388 | 5.1 | 6.14 | 1.65 | 0.47 | 1.68 |
| 74 | IG74929 | 4.9 | 8.29 | 2.39 | 0.41 | 2.65 |
| 75 | IG74940 | 5.1 | 8.76 | 2.08 | 0.35 | 2.09 |
| 76 | IG75406 | 5.0 | 9.89 | 1.86 | 0.34 | 2.00 |
| 77 | IG7722 | 4.7 | 10.70 | 1.92 | 0.27 | 1.97 |
| 78 | IG9239 | 4.7 | 9.93 | 1.98 | 0.38 | 2.20 |
| 79 | IG9244 | 4.8 | 7.18 | 2.43 | 0.48 | 2.60 |
| 80 | IG9413 | 5.6 | 10.46 | 2.90 | 0.49 | 3.03 |
| 81 | IG9433 | 5.4 | 7.98 | 1.93 | 0.36 | 2.11 |
| 82 | IG9434 | 5.2 | 7.48 | 2.46 | 0.41 | 2.56 |
| 83 | IG9438 | 5.0 | 8.92 | 1.74 | 0.30 | 1.83 |
| 84 | IG9586 | 4.8 | 7.98 | 2.11 | 0.33 | 2.20 |
| 85 | IG117696 | 5.5 | 10.43 | 1.75 | 0.3 | 1.81 |
| 86 | IG117697 | 5.1 | 7.58 | 1.58 | 0.24 | 1.54 |
| 87 | IG117699 | 5.6 | 7.65 | 1.15 | 0.14 | 1.62 |
| 88 | IG117700 | 5.5 | 8.34 | 1.42 | 0.29 | 1.55 |
| 89 | IG117714 | 5.9 | 9.94 | 0.6 | 0.17 | 0.71 |
| 90 | IG117718 | 5.5 | 7.45 | 1.51 | 0.29 | 1.51 |
| 91 | IG125018 | 4.4 | 10.3 | 0.88 | 0.15 | 0.86 |
| 92 | IG125021 | 4.9 | 8.13 | 1.66 | 0.26 | 1.73 |
| 93 | IG125028 | 4.8 | 9.3 | 1.21 | 0.24 | 1.3 |
| 94 | IG128430 | 4.5 | 13.51 | 0.74 | 0.14 | 0.75 |
| 95 | IG128434 | 4.6 | 10.2 | 1.38 | 0.29 | 1.36 |
| 96 | IG128506 | 4.8 | 10.11 | 1.22 | 0.28 | 1.17 |
| 97 | IG131985 | 4.6 | 7.52 | 1.38 | 0.23 | 1.4 |
| 98 | IG6050 | 5.3 | 6.73 | 1.31 | 0.22 | 1.44 |
| 99 | IG6057 | 5.7 | 9.65 | 1.62 | 0.36 | 1.74 |
| 100 | IG6111 | 5.3 | 8.98 | 1.28 | 0.24 | 1.45 |
| 101 | IG6446 | 4.6 | 9.19 | 1.28 | 0.22 | 1.37 |
| 102 | IG6468 | 5.2 | 13.66 | 1.77 | 0.32 | 1.9 |
| 103 | IG69605 | 5 | 8.51 | 1.75 | 0.24 | 1.64 |
| 104 | IG69620 | 5.7 | 6.95 | 1.68 | 0.33 | 1.76 |
| 105 | IG70277 | 5.7 | 7.54 | 1.5 | 0.23 | 1.47 |
| 106 | IG70286 | 5.4 | 10.29 | 1.8 | 0.32 | 2.09 |
| 107 | IG70294 | 5.7 | 10.99 | 1.57 | 0.26 | 1.57 |
| 108 | IG70297 | 5.9 | 5.24 | 1.56 | 0.24 | 1.57 |
| 109 | IG70305 | 5.4 | 5.92 | 1.52 | 0.32 | 1.62 |
| 110 | IG70311 | 5.2 | 9.68 | 1.21 | 0.23 | 1.41 |
| 111 | IG70312 | 4.7 | 9.18 | 1.23 | 0.22 | 1.41 |
| 112 | IG70328 | 5.4 | 7.82 | 1 | 0.14 | 1.07 |
| 113 | IG70330 | 5.5 | 7.16 | 1.39 | 0.23 | 1.46 |
| 114 | IG70334 | 5.7 | 6.49 | 2.18 | 0.18 | 1.6 |
| 115 | IG70345 | 6 | 7.91 | 1.75 | 0.26 | 1.65 |
| 116 | IG70346 | 5.6 | 5.56 | 1.47 | 0.18 | 1.36 |
| 117 | IG70347 | 5.8 | 6.35 | 1.4 | 0.32 | 1.5 |
| 118 | IG70348 | 5.2 | 6.65 | 1.39 | 0.28 | 1.38 |
| 119 | IG70349 | 5.5 | 8.81 | 1.47 | 0.2 | 1.48 |
| 120 | IG70351 | 5.4 | 6.92 | 1.45 | 0.24 | 1.55 |
| 121 | IG70357 | 6.1 | 8.95 | 1.65 | 0.31 | 1.78 |
| 122 | IG70361 | 4.5 | 13.85 | 1.31 | 0.22 | 1.38 |
| 123 | IG70363 | 5.6 | 8.78 | 1.33 | 0.22 | 1.39 |
| 124 | IG70369 | 5.8 | 6.53 | 1.41 | 0.19 | 1.47 |
| 125 | IG70370 | 5.6 | 7.63 | 1.95 | 0.32 | 1.86 |
| 126 | IG70375 | 5.5 | 6.08 | 1.08 | 0.18 | 1.22 |
| 127 | IG70377 | 4.4 | 8.7 | 1.51 | 0.28 | 1.51 |
| 128 | IG70381 | 5.3 | 7.09 | 0.82 | 0.16 | 0.82 |
| 129 | IG70383 | 5.5 | 6.44 | 1.42 | 0.3 | 1.64 |
| 130 | IG70388 | 5.2 | 8.26 | 1.19 | 0.21 | 1.12 |
| 131 | IG70389 | 4.2 | 10.49 | 1.5 | 0.27 | 1.54 |
| 132 | IG70390 | 5.9 | 6.42 | 1.28 | 0.2 | 1.24 |
| 133 | IG70402 | 4.9 | 10.89 | 1.65 | 0.21 | 1.62 |
| 134 | IG70408 | 4.8 | 9.16 | 1.56 | 0.24 | 1.59 |
| 135 | IG70409 | 4.7 | 8 | 1.47 | 0.25 | 1.55 |
| 136 | IG70410 | 5.4 | 10.57 | 1.2 | 0.25 | 1.22 |
| 137 | IG70413 | 5 | 9.57 | 1.16 | 0.18 | 1.23 |
| 138 | IG70416 | 4.7 | 7.78 | 1.6 | 0.24 | 1.63 |
| 139 | IG70419 | 4.8 | 6.88 | 1.21 | 0.14 | 1.08 |
| 140 | IG70421 | 4.4 | 9.68 | 0.92 | 0.14 | 0.78 |
| 141 | IG70762 | 5.7 | 7.21 | 1.52 | 0.28 | 2.16 |
| 142 | IG70773 | 4.3 | 8.73 | 1.38 | 0.22 | 1.48 |
| 143 | IG70774 | 5.7 | 7.66 | 1.53 | 0.29 | 1.52 |
| 144 | IG70775 | 5.1 | 4.75 | 0.75 | 0.16 | 0.69 |
| 145 | IG70778 | 4.9 | 8.57 | 1.22 | 0.2 | 1.14 |
| 146 | IG70780 | 5.3 | 9.72 | 1.76 | 0.28 | 1.52 |
| 147 | IG70783 | 4.8 | 10.5 | 1.18 | 0.21 | 1.27 |
| 148 | IG70784 | 5.7 | 8.97 | 1.57 | 0.31 | 1.73 |
| 149 | IG70787 | 4.9 | 8.12 | 0.81 | 0.15 | 1 |
| 150 | IG70789 | 5.3 | 7 | 1.05 | 0.17 | 1.01 |
| 151 | IG70822 | 6.3 | 9.3 | 0.78 | 0.17 | 0.79 |
| 152 | IG73369 | 5.7 | 8.4 | 1.4 | 0.22 | 1.46 |
| 153 | IG73381 | 6.2 | 9.28 | 1.48 | 0.27 | 1.8 |
| 154 | IG73382 | 5.3 | 8.61 | 0.98 | 0.15 | 0.95 |
| 155 | IG73386 | 5.8 | 8.61 | 1.23 | 0.2 | 1.21 |
| 156 | IG73390 | 5.3 | 11.8 | 1.89 | 0.34 | 1.82 |
| 157 | IG74021 | 5.6 | 9.76 | 1.29 | 0.24 | 1.33 |
| 158 | IG74994 | 5.8 | 6.99 | 1.25 | 0.21 | 1.28 |
| 159 | IG74995 | 4.7 | 8.61 | 1.25 | 0.25 | 1.35 |
| 160 | IG75029 | 4.4 | 8.58 | 1.18 | 0.18 | 1.09 |
| 161 | IG75360 | 5.6 | 8.47 | 1.09 | 0.19 | 1.1 |
| 162 | IG7714 | 6.1 | 6.82 | 1.23 | 0.25 | 1.26 |
| 163 | IG7717 | 4.9 | 5.92 | 1.15 | 0.21 | 1.22 |
| 164 | IG7758 | 5.2 | 10.01 | 1.41 | 0.29 | 1.47 |
| 165 | IG8447 | 5.4 | 7.3 | 0.88 | 0.16 | 1.02 |
| 166 | IG8914 | 5.5 | 6.62 | 1.03 | 0.18 | 1.07 |
| 167 | IG9088 | 5.2 | 12.63 | 1.69 | 0.35 | 1.8 |
| 168 | IG9425 | 4.8 | 8.81 | 1.48 | 0.26 | 1.54 |
| 169 | IG9430 | 5.8 | 10.74 | 1.57 | 0.28 | 1.68 |
| 170 | IG9431 | 5.8 | 6.96 | 1.22 | 0.25 | 1.42 |
| 171 | IG9435 | 5.3 | 12.59 | 1.78 | 0.31 | 1.91 |
| 172 | IG9436 | 5.3 | 8.81 | 1.55 | 0.29 | 1.63 |
| 173 | IG9437 | 4.8 | 15.23 | 1.7 | 0.26 | 1.59 |
| 174 | IG9439 | 4.4 | 7.63 | 1.14 | 0.18 | 1.15 |
| 175 | IG9440 | 5.5 | 7 | 1.33 | 0.25 | 1.35 |
| 176 | IG9441 | 6.5 | 6.98 | 1.46 | 0.21 | 1.41 |
| 177 | IG9442 | 5.7 | 8.28 | 0.99 | 0.21 | 1.07 |
| 178 | IG9584 | 6.1 | 8.31 | 1.19 | 0.17 | 1.35 |
| 179 | IG9627 | 5 | 9.91 | 1.43 | 0.21 | 1.54 |
| 180 | IG9629 | 4.6 | 8.39 | 1.5 | 0.26 | 1.56 |
| 181 | IG9630 | 5.1 | 13.43 | 1.47 | 0.25 | 1.43 |
| 182 | howzat | 5.4 | 9.08 | 0.48 | 0.15 | 0.43 |
| 183 | pbahattrick | 5.3 | 11.34 | 0.75 | 0.14 | 0.82 |
| 184 | pbapistol | 5.5 | 10.22 | 1 | 0.14 | 1 |
| 185 | Pbabaumary | 5.3 | 10.04 | 0.81 | 0.14 | 0.93 |
| 186 | IG115380 | 7.4 | 5.13 | 0.76 | 0.12 | 0.69 |
| 187 | IG70309 | 7.1 | 4.33 | 0.51 | 0.13 | 0.62 |
| 188 | IG115390 | 7.3 | 6.69 | 0.93 | 0.20 | 0.95 |
| 189 | IG117708 | 6.7 | 5.69 | 1.07 | 0.19 | 1.14 |
| 190 | IG131983 | 5.7 | 6.64 | 0.73 | 0.14 | 0.67 |
| 191 | IG134564 | 6.5 | 6.14 | 0.90 | 0.23 | 0.99 |
| 192 | IG6041 | 5.9 | 6.45 | 0.94 | 0.16 | 0.94 |
| 193 | IG70275 | 5.8 | 6.67 | 0.97 | 0.17 | 0.96 |
| 194 | IG70280 | 6.2 | 6.69 | 0.96 | 0.15 | 0.91 |
| 195 | IG70291 | 6.5 | 4.82 | 0.88 | 0.17 | 0.96 |
| 196 | IG70299 | 6.1 | 4.65 | 0.50 | 0.09 | 0.56 |
| 197 | IG70308 | 6.4 | 5.55 | 1.02 | 0.17 | 0.95 |
| 198 | IG70374 | 6.4 | 5.53 | 1.36 | 0.17 | 1.35 |
| 199 | IG70760 | 6.4 | 6.50 | 1.03 | 0.09 | 0.87 |
| 200 | IG70761 | 6.5 | 4.85 | 1.24 | 0.22 | 1.24 |
| 201 | IG70768 | 6.7 | 3.31 | 0.63 | 0.09 | 0.54 |
| 202 | IG70788 | 6.3 | 5.42 | 1.06 | 0.23 | 1.05 |
| 203 | IG74979 | 7.0 | 4.53 | 1.33 | 0.24 | 1.33 |
| 204 | IG8985 | 6.0 | 5.44 | 0.52 | 0.10 | 0.61 |
